# The predictive role of coping styles and sense of coherence in the post-traumatic growth of mothers with disabled children: a cross-sectional study

**DOI:** 10.1186/s12888-022-04357-5

**Published:** 2022-11-15

**Authors:** Akram Farhadi, Masoud Bahreini, Ainaz Moradi, Kamran Mirzaei, Reza Nemati

**Affiliations:** 1 The Persian Gulf Tropical Medicine Research Center, The Persian Gulf Biomedical Sciences Research Institute, Bushehr University of Medical Sciences, Bushehr, Iran; 2 Department of Nursing, School of Nursing and Midwifery, Bushehr University of Medical Sciences, Bushehr, Iran; 3 Student Research Committee, Bushehr University of Medical Sciences, Bushehr, Iran; 4 Department of Community Medicine, School of Medicine, Bushehr University of Medical Sciences, Bushehr, Iran; 5 Department of Nursing, School of Nursing and Midwifery, Bushehr University of Medical Sciences, Bushehr, Iran

**Keywords:** Coping style, Disability, Emotion-focused, Post-traumatic growth, Sense of coherence

## Abstract

**Background:**

Coping and accepting stressful events can lead to positive psychological changes, growth, and excellence. In this regard, the present study was conducted to determine the relationship between coping styles and sense of coherence with post-traumatic growth in mothers with disabled children in Bushehr (a southern city in Iran).

**Methods:**

The present cross-sectional study was performed on 260 mothers with children with disabilities who were filed in the rehabilitation centers covered by the Welfare Department of Bushehr in 2018. Demographic information form, Tedeschi and Calhoun post-traumatic growth questionnaire, Antonovsky sense of coherence, and Billings and Moos coping styles were employed to collect data. Data were analyzed by SPSS software using Pearson correlation coefficient and linear regression analysis at a significance level of less than 0.05.

**Results:**

The mean age and standard deviation were 35.83 ± 7.41 for the mothers, and 7.20 ± 4.05 for the children. The mean and standard deviation of post-traumatic growth was 64.88 ± 14.90, sense of coherence was 116.36 ± 22.65 and coping styles was 30.59 ± 6.39. The results of linear regression show that only the two dimensions of meaningfulness (*p* = 0.013 and β = 0.170) and manageability from the sense of coherence (*p* = 0.001 and β = 0.432) can predict post-traumatic growth in mothers with disabled children in Bushehr. Also, from the coping style dimensions, only the emotion-focused variable (*p* = 0.001 and β = 0.353) is a predictor of post-traumatic growth.

**Conclusion:**

Considering the role of sense of coherence and coping style in predicting post-traumatic growth, to implement rehabilitation programs and support the families of these children, it is essential to plan for the development of social and psychological support for mothers with disabled children.

## Background

The phenomenon of disability is an undeniable reality and one of the most complex and difficult issues for children and adolescents in today’s society, which persists into adulthood. According to global statistics, there are more than one billion people with disabilities in the world [1]. In Iran, according to the Welfare Organization, nearly 7% of the population suffers from at least one type of disability [2].

The birth of a child is a source of stress for parents of all ages and circumstances. More specifically, the birth and presence of a child with a disability has a deeper and greater impact on the family, and the psychological, social, and economic pressures resulting from the presence of such a child in the family are several times greater than a healthy child [3, 4]. Raising children with disabilities poses many challenges. The parents of these children face long-term care, additional medical expenses, and feeling of shame, and they also experience physical and psychological conflicts due to the special care of the child [5]. In addition, this issue can damage the coherence and structure of the family so that it can lead to changes in family functioning and adjustment [6, 7].

In most societies, mothers usually play a more active role in caring for their sick children compared to other family members [8]. The mother is the first person to have direct contact with the child, and in dealing with her disabled child, she experiences different emotions such as feeling guilty, feeling failure in achieving life goals, and sometimes in response to this nervous tension, they give extreme support for the children [9, 10].

However, the results of some studies have shown that these stressful events may act as a facilitator for positive psychological changes and in the presence of stressful living conditions, growth is also observed [11–13]. In other words, although a stressful accident affects different aspects of a person’s life negatively, coping with this accident can also lead to growth. Experience or mental perception of positive psychological changes caused by a conflict with stressful events is termed “post-traumatic growth”. In this context, Tedeschi and Calhoun (1996) have identified five dimensions for it, including new situations, interpersonal relationships, appreciation for life, personal strength, and spiritual change [14]. Zwahlen et al. (1996) also pointed out that post-traumatic growth is not directly the result of trauma, but rather it is the coping strategy that is adopted in dealing with trauma that determines post-traumatic growth [15]. When a disabled child is born, the mother tries to cope with this stressful event. The ability of a person to deal with stressors can be examined from two aspects. The first aspect is problem-focused coping strategies including an active confrontation with the problem, planning, refraining from competitive activities and hasty actions, and seeking social support in coping with the stressful situation. The second aspect is the emotion-focused coping strategies including no mental involvement with the problem, denial, no behavioral involvement to solve the problem and focus on emotion [16]. Reports indicate that individuals adopt any form of confrontation to fight against most stressful events [9].

One of the factors related to how a person cope with stress is the personal attitude towards life or sense of coherence. The idea was first proposed by Aaron Antonovsky, and he asserted that control over stress is done through three basic concepts, comprehensibility, manageability, and meaningfulness of the events from the individual’s point of view [17]. There is disagreement about the predictive role of a sense of coherence on the post-traumatic growth variable in studies [18]. While the study of Xiu et al. (2018) confirms a positive relationship [19], others have found no significant correlation between the two [20, 21]. With these explanations, the necessity of repeating such a study to examine the relationship between a sense of coherence and post-traumatic growth can be emphasized.

For thousands of years, the concept of post-traumatic growth in confronting life’s difficulties and crises has been considered in various religions [22]. Due to the dependence of the post-traumatic growth structure on the cultural context and religious characteristics [23–25] and the differences in religion and culture between Western and Middle Eastern societies, the results of studies in other parts of the world with different contexts may not be generalizable in the Middle Eastern societies. Iranians, with their own cultural and historical characteristics, have different concerns and beliefs on the subject of disability [26]. According to the explanation given regarding the importance of coping styles and sense of cohesion against stressors, the predictive role of these variables on post-traumatic growth is significant.

### Aim of the study

The study aimed to investigate the relationship between coping styles and sense of coherence in the post-traumatic growth of mothers with disabled children.

## Methods

### Design, settings, and participants

The present research is a cross-sectional study. The study population included mothers with disabled children in Bushehr (North of Persian Gulf) who were filed in the Welfare Department. The research environment included all public and private rehabilitation centers under the supervision of the Bushehr Welfare Department, and the data was collected there in a period of 3 months (May to August 2018). Finally, seven rehabilitation centers were selected. To decide on the sample size, according to Zhang et al. (2013) [27], and the relationship between post-traumatic growth and coping styles *r* = 0.553, we assumed a moderate correlation between post-traumatic growth, a sense of coherence *r* = 0.5, α = 0.05, and power = 85%. Then, using the formula below, with a calculation of 10% loss, the sample size was determined to be 52 disabled people.

$$n=\left[\frac{Z_{1-\frac\propto2}+Z_{1-\beta}}C\right]^2+3$$

$$C=0.5ln\;\left(\frac{1+r}{1-r}\right)$$

Concerning the five disability groups who were observed and compared, the total sample size was *N* = n×$\sqrt{4}$, the total volume was estimated to be 260 people (each group of disability is approximately 52 people). Samples were selected by simple random sampling method from among the patients filed in Bushehr rehabilitation centers. Inclusion criteria included informed consent to participate in the study, having a child with a disability (mental, physical-motor, sensory, autism, multiple disabilities), considered mild, moderate, severe, and very severe disability confirmed by the Medical Commission based on the International Classification of Functioning, Disability, and Health (ICF), having experienced at least 6 months of care for a disabled child, being the primary caregiver of a child with a disability, and not suffering from any severe psychiatric disorder including schizophrenia, major depression, and bipolar disorder (self-reported).

### Data quality control

The researcher randomly selected the participants from the list of mothers with disabled children based on the inclusion criteria in the welfare centers. After explaining the objectives of the research and obtaining informed consent, study questionnaires were delivered to them for completion. For illiterate mothers, the questionnaire was read to them by the researcher, and it was completed based on their responses.

### Data collection procedure and instruments

Data collection tools included a demographic information form and post-traumatic growth questionnaires of Tedeschi and Calhoun, Billings and Moos coping style, and Aaron Antonovsky sense of coherence. Demographic information of mothers included age, number of children, marital status, number of years of education, occupation, economic status, length of childcare period and number of disabled children in the family, and demographic form of children included age, gender, type of disability, severity and the cause of disability.

#### Post-Traumatic Growth Inventory (PTGI)

The Post-Traumatic Growth Inventory (PTGI) introduced by Tedeschi and Calhoun (1996) has 21 items that have 5 domains (new situations, interpersonal relationships, appreciation for life, personal strength, and spiritual change) determine the rate of psychological development after dealing with a stressful accident. This instrument is scored on a 6-point Likert scale, with the first option (no) scoring zero and the second to sixth options scoring 1 to 5. The range of scores is from zero to 105, and a higher score indicates more growth while a lower score indicates less growth [14]. In a study by Tedeschi and Calhoun (1996), the overall alpha coefficient of the questionnaire was 0.90. The Cronbach’s alpha range for each subscale was between 0.67 and 0.85 [14]. The validity and reliability of this tool in Iran were confirmed by Najafi Gharehasani et al. (2020) and the total Cronbach’s alpha coefficient was set to 0.94 [28]. In the present study, Cronbach’s alpha coefficient for PTGI was 0.89.

#### Antonovsky sense of coherence questionnaire

Antonovsky Sense of Coherence Questionnaire (1987) contains 29 questions, the answer to each question is set on a 7-point Likert scale from a score of 1 (never) to a score of 7 (always). The dimensions of this questionnaire include comprehensibility, manageability, and meaningfulness. The overall score for each person is between 29 and 203 [29]. Higher scores indicate a greater sense of cohesion. The validity and reliability of the questionnaire in Iran were confirmed by Aghayousefi and Sharif (2011), Cronbach’s alpha coefficient was 0.96 [30]. In the present study, Cronbach’s alpha coefficient for this questionnaire was 0.82.

#### Coping Strategies Questionnaire (CSQ)

The Coping Strategies Questionnaire by Billings and Moos (1981) consists of 19 questions that assess emotion-focused coping styles with 8 items and problem-focused coping styles with 11 items [31]. In this questionnaire, a four-choice Likert scale was used to score the items, including always = 4, often = 3, sometimes = 2, and never = 1. The total score of coping strategies for each person was between 19 and 76. The score of the problem-focused coping style is between 11 and 44, and the score of the emotion-focused coping style is between 8 and 32. Questions 3, 4, 6, 9, 12, 15, 18, and 19 are related to the field of emotion-focused and the rest (eleven questions) are related to the field of problem-focused opposites. A higher score on each subscale means that the person has applied more problem-focused or emotion-focused coping methods. Araghian Mojarad et al. (2020) obtained the validity and reliability of this questionnaire by internal consistency method through Cronbach’s alpha of 0.80 [32]. In the present study, Cronbach’s alpha coefficient for coping strategies questionnaire was 0.71.

### Ethical consideration

This study was reviewed and approved by the ethics committee of Bushehr University of Medical Sciences and has the ethics code IR.BPUMS.REC.1398.019. The participants were also told that participation in the study was optional and that their names would not be included in the questionnaire due to the confidentiality of the individuals’ information. In addition, informed consent was obtained from the participants under the Helsinki Accords.

### Data analysis

Descriptive statistics (mean, standard deviation, frequency, and percentage) and inferential statistics (Pearson correlation coefficient, Spearman correlation, and linear regression) were used to analyze the data using SPSS software version 23 (SPSS Inc., Chicago, IL, USA). The significance level was considered less than 0.05. The normality of data distribution was confirmed by Kolmogorov-Smirnov statistical method.

## Results

### Sociodemographic characteristics

In total, 260 mothers with disabled children were studied this research. Of these, 94.6% were married, 93.8% were housewives, and 67.3% were in moderate economic conditions. Most of the disabled children (58.1%) were male, and in terms of the type of disability, 28.1% had a physical disability, 40.8% had moderate severity of the disability, and 65.8% had disabilities with congenital cause. The mean and standard deviation of the age of mothers participating in the study and their children with disabilities were 35.83 ± 7.41 and 7.20 ± 4.05 years, respectively (Table 1).

**Table 1:** Demographic Characteristics of Mothers and Children with Disabilities (*n* = 260)

Variables		Subgroup	Number	Frequency
**Mothers**	Marital status	Married	246	94.6
		Divorced	9	3.5
		Widow	5	1.9
	Education	Illiterate	12	4.6
		Primary school	65	25
		Middle school	53	20.4
		High school	92	35.4
		University	38	14.3
	Job	Housekeeper	244	93.8
		Retired	1	0.4
		Employee	11	4.3
		Freelance	4	1.5
	Economic status	Good	28	10.8
		Medium	175	67.3
		Weak	57	21.9
**Children**	Gender	Girl	109	41.9
		Boy	151	58.1
	Severity disability	Light	51	19.6
		Medium	106	40.8
		Severe	86	33.1
		Very severe	17	6.5
	Cause of disability	Congenital	171	65.8
		Adventitious	89	34.2
	Type of disability	Mental	53	20.4
		Physical-motor	73	28.1
		Sense (auditory-speech-visual)	52	20
		Autism	25	9.6
		Multiple disabilities	57	21.9

### Mean and standard deviation of post-traumatic growth, sense of coherence, and coping styles

The results showed that all mothers with disabled children participating in this study showed some degree of growth, and the mean and standard deviation of the post-traumatic growth score was 64.88 ± 14.90. The highest and lowest mean score expressions in different dimensions were obtained for the dimensions of spiritual change (3.28 ± 2.53) and new situations (2.96 ± 4.24). The findings also showed that the mean and standard deviation of the total score of sense of coherence is 116.36 ± 22.65. Also, most of them adopted problem-focused coping strategies (Table 2).

**Table 2:** Mean and Standard Deviation of Post-Traumatic Growth, Sense of Coherence, and Coping Styles in Mothers with Disabled Children (*n* = 260)

Variables	Subgroup	Mean	Standard Deviation	Minimum	Maximum
**Post-Traumatic Growth**	Interpersonal Relationships	21.43	5.82	6	35
	New Situations	14.83	4.24	5	25
	Personal Strength	12.80	3.54	4	20
	Spiritual Change	6.56	2.53	0	10
	Appreciation for Life	9.24	2.69	2	15
	Total Post-Traumatic Growth Score	64.88	14.90	25	101
**Sense of Coherence**	Comprehensibility	41.04	9.96	6	69
	Manageability	40.72	8.39	16	63
	Meaningfulness	34.00	9.31	8	56
	Total Score of Sense of Cohesion	116.36	22.65	47	181
**Coping Styles**	Problem-Focused Coping Styles	18.23	9.96	6	69
	Emotion-Focused Coping Styles	12.36	8.39	16	63
	The Total Score of the Coping Style	30.59	9.31	8	56

### Correlation of post-traumatic growth with a sense of coherence and coping styles

The results of the correlation matrix showed that there is a significant direct linear relationship between the total score of post-traumatic growth and all its dimensions with the sense of coherence and coping style. This correlation is also stronger in the dimension of “Meaningfulness” (Table 3).

**Table 3:** Correlation Coefficients Between Variables (*n* = 260)

Dimensions of Variables	1	2	3	4	5	6	7	8	9	10	11	
**1. Problem-Focused Coping Styles**	1											
**2. Emotion-Focused Coping Styles**	0.498^b^	1										
**3. Comprehensibility**	-0.267^b^	0.044	1									
**4. Manageability**	-0.091	0.202^b^	0.484^b^	1								
**5. Meaningfulness**	0.024	0.165^b^	0.441^b^	0.588^b^	1							
**6. Interpersonal Relationships**	0.144^a^	0.334^b^	0.316^b^	0.363^b^	0.375^b^	1						
**7. New Situations**	0.170^b^	0.323^b^	0.196^b^	0.377^b^	0.532^b^	0.579^b^	1					
**8. Personal Strength**	0.213^b^	0.294^b^	0.289^b^	0.425^b^	0.458^b^	0.515^b^	0.657^b^	1				
**9. Spiritual Change**	0.140^a^	0.217b	0.279^b^	0.280^b^	0.327^b^	0.494^b^	0.399^b^	0.424^b^	1			
**10. Appreciation for Life**	0.317^b^	0.297^b^	0.142^a^	0.328^b^	0.469^b^	0.438^b^	0.592^b^	0.577^b^	0.380^b^	1		
**11. Post-Traumatic Growth**	0.236^b^	0.383^b^	0.321^b^	0.456^b^	0.547^b^	0.841^b^	0.842^b^	0.802^b^	0.646^b^	0.723^b^	1	

^a^ Correlation is significant at the 0.05 level (2-tailed)

^b^ Correlation is significant at the 0.01 level (2-tailed)

### The predictive role of sense of coherence and coping styles in post-traumatic growth

The results of linear regression show only dimensions of meaningfulness (*p* = 0.013 and β = 0.170) and manageability from the sense of coherence (*p* = 0.001 and β = 0.432) can predict post-traumatic growth in mothers with disabled children in Bushehr. The results of the analysis indicate that the sense of coherence accounts for only 30% of post-traumatic growth changes in mothers with disabled children (Adjusted *R*^2^ = 0.33). Also, based on the results of the linear regression coefficient model, from the coping style dimension, only the emotion-focused variable (*p* = 0.001 and β = 0.353) is a predictor of post-traumatic growth. The results of the analysis indicate that the sub-domains of coping style explain only 14% of post-traumatic growth changes in mothers with disabled children (Adjusted *R*^2^ = 0.143) (Table 4).

**Table 4:** Predictive Power of Sense of Cohesion and Coping Styles in Post-Traumatic Growth Styles in Mothers with Disabled Children (*n* = 260)

Variables	Subgroup	B	Std. Error	β	*P* value	95.0% Confidence Interval for B	
						**Lower Bound**	**Upper Bound**
**Sense of Cohesion**	Manageability	0.306	0.123	0.170	0.013	0.064	0.548
	Meaningfulness	0.699	0.110	0.432	≥ 0.001	0.483	0.914
	Comprehensibility	0.104	0.096	0.068	0.280	-0.085	0.294
**Coping Styles**	Problem-Focused Coping Styles	0.207	0.227	0.061	0.362	-0.239	0.654
	Emotion-Focused Coping Styles	1.763	0.332	0.353	≥ 0.001	1.110	2.416

## Discussion

This study was conducted to identify whether post-traumatic growth in mothers of children with disabilities is related to their coping style and sense of family cohesion. The present study showed that post-traumatic growth has a positive and significant correlation with all sub-dimensions of copying styles and the sense of cohesion. The results also showed that emotion-oriented coping style plays a significant role in predicting post-traumatic growth in mothers having children with disabilities.

Previous studies in the field of mothers’ care for children with disabilities focused on psychological disorders and the negative consequences of stress, while recent studies have focused on the positive effects of being in stressful situations in other groups. Therefore, investigating post-traumatic growth and related factors such as coping style and feeling of cohesion in this group seems significant and necessary, which is one of the strengths of the present study.

### Investigating the dimensions of post-traumatic growth

According to the results of the present study, all mothers had some degree of post-traumatic growth. These results are in line with the results of some other studies in other groups such as mothers with children with cancer and autism who have experienced struggling with stressful situations. For example, the results of the study of Heidarzadeh et al. (2015) conducted in Iran showed that despite numerous physical, psychological, and social problems in cancer patients, exposure to stressful events leads to positive psychological consequences and the patients grow post-traumatically [33]. These results are supported by other studies such as Lu et al. (2022) [34], Qin et al.(2021) [35], Morris et al. (2020), Zebrack et al. (2015), and Zhang et al. (2013) [27, 36, 37]. On the other hand, the results of the study by Yonemoto et al. (2011), which was performed on parents with children with cancer, showed that they experienced a lower average growth rate [38], which is inconsistent with the results of the present study. Given that cultural and social factors can affect the concept of post-traumatic growth, differences in the context of the study and the nature of the stress that parents were involved in may cause the disagreement between the results of that study and the present study.

The present study showed that among the dimensions of post-traumatic growth, the highest average was related to spiritual dimensions. Among the studies with consistent results, Heidarzadeh et al. (2015) in a combined study on the concept of post-traumatic growth in cancer survivors in Iran showed that spiritual growth is observed in both domains of intellectual beliefs (with the theme of self-return, seeking for the meaning of life, inner peace, deepening of spiritual beliefs, acceptance of death and closeness to God) and practical beliefs (with the themes of self-cultivation and highlighting positive attributes) [33]. Regarding the religious and traditional beliefs which are common especially among Iranian women [39], who always view the hardships as a divine test and in this way expect the rewards in the hereafter from God, it is acceptable that they demonstrate a higher mean of spiritual growth than the other dimensions of post-traumatic growth. In some studies, such as Mystakidou et al. (2008), Manne et al. (2004), Cordova et al. (2001) [40–42] spiritual growth had the lowest mean among the dimensions of spiritual growth. The difference in the context of these studies in comparison with studies conducted in Asian countries such as Iran can justify the differences in findings [39, 43].

### Investigating the mean of sense of coherence

The results of coherence assessment in this study showed that all mothers with children with disabilities have a moderate level of coherence. In this regard, Avaznejad et al. (2017) in a study aimed at comparing the sense of coherence in mothers with healthy children and mothers with children with chronic diseases showed that the total mean of sense of coherence was at a moderate level in the group of mothers [44] which is consistent with the results of the present study. However, Brockhouse et al. (2011) in a study entitled “Determining the relationship between sense of coherence, social support and empathy on post-traumatic growth” achieved results inconsistent with the present study. In fact, the results of their study showed a weak mean of coherence [21]. In explaining the inconsistent results with the results of the present study, it can be stated that a weak sense of coherence can be due to factors such as lack of social and emotional support and less ability to cope with stressors.

### Investigating the mean of coping styles

The results of this study showed that the average rate of problem-focused coping styles in mothers with children with disabilities is higher than the average rate of emotion-focused coping styles. In this regard, Ehteshamzadeh et al. (2013) conducted a study on the components of coping style and sense of coherence and found that the mean score of problem-focused coping style was higher than emotion-focused coping style [45]. Also, the study of Ghasemi and Hadianfard (2014), as well as the study of Askari et al. (2011), which examined the components of coping style among addicted and healthy men, showed that the control group had a higher mean in problem-focused coping style than the emotion-focused one, which confirms the results of the present study [46, 47]. However, the study by Zandi Karimi (2016), which measured the effectiveness of group spiritual therapy on resilience and coping styles of cancer patients, showed that the control group had a higher mean in emotion-focused coping style than the problem-focused one [48]. The findings of Lin et al. (2011) showed that applying problem-focused coping strategies is associated with greater adaptation and flexibility of parents [49].

### The relationship between post-traumatic growth and coping styles

Results of this study also indicated that post-traumatic growth has a positive and significant relationship with coping style and all sub-dimensions of coping style. The emotion dimension also has a stronger correlation with post-traumatic growth. The results of the study of Wilson et al. (2016) are in line with the results of the present study. Their study showed that there is a direct relationship between post-traumatic growth in mothers having children with cancer and a positive coping style [50]. Some results of Zhang et al.‘s (2013) study supports the results of the present study while other results of that study contradict the results of the present study. In the study by Zhang et al., the positive coping style which is relatively in line with problem-focused styles is directly related to post-traumatic growth, but the negative coping style, which is more in line with emotional coping styles, was not significantly related to post-traumatic growth [27]. In the studies by Wilson et al. (2016), Taghavi et al. (2015), Thombre et al. (2010) and Prati et al. (2009) religious confrontation is mentioned as a factor of post-traumatic growth [50–53]. However, in another study, Mehrabi et al. (2015) concluded that post-traumatic growth had a positive and significant relationship with problem-focused coping style, but it had no relationship with avoidant coping style [54] and therefore, their findings did not agree with the results of the present study. The reason behind this discrepancy seems to be the introduction of emotional coping styles as avoidance or negative coping style. The results of the present study also showed that religious confrontation is another form of emotional coping, that is, it is related to the dimension of spiritual changes in post-traumatic growth, which is not far from the results of other research [55]. This is because, despite the use of the problem-focused coping style, mothers of children with disabilities more often turn to emotion-focused coping styles, which may be due to the high level of stress experienced by them. As the results of the present study showed, mothers with disabled children experience spiritual growth. Therefore, one of the implications of these results can be seen in the study by Vollrath et al. (1995) [55], in which they advocated positive emotion-coping style as another form of spiritual change (spirituality). However, in studies related to religion and psychology, conflicting results concerning post-traumatic growth have been obtained, that is, some studies have reported negative correlations [56] and some others have reported positive correlations between religious beliefs and post-traumatic growth [57].

### The relationship between post-traumatic growth and a sense of coherence

Results of this study disclosed a positive and significant relationship between post-traumatic growth and a sense of coherence and all its sub-dimensions. In addition, the sub-domain of meaningfulness has a stronger relationship with post-traumatic growth. The results of Lopez et al.‘s (2015) study showed that a sense of coherence is effective in improving post-traumatic growth [58]. Also, the results of the study of Forstmeier et al. (2010) and Almedom et al. (2005) showed that there is a positive and significant relationship between the sense of coherence and post-traumatic growth, which is consistent with our results [59, 60]. But Brockhouse et al. (2011), in a study aimed at determining the effect of sense of coherence, and social support and empathy on post-traumatic growth, achieved results inconsistent with the present study [19]. The results of the study by Brockhouse et al. (2011) showed that the sense of coherence has a significant negative relationship with post-traumatic growth. Explaining the inconsistent results with the results of the present study, it can be claimed that a weak sense of coherence can be caused by factors such as lack of social and emotional support and the individual’s inability in dealing with stressors [21].

### Predictability of post-traumatic growth

In terms of predictability, the results of this study showed that emotional coping style plays a role in predicting post-traumatic growth in mothers with children with disabilities. The results of the study by Svetina et al. (2012) mentioned that coping style predicts post-traumatic growth, and therefore, it was consistent with our results [61]. Other consistent studies include the study by Prati et al. (2009), who found that positive emotional coping (religious coping) is a predictor of post-traumatic growth [53], and the study by Zhang et al. (2011), who stated that positive coping style is predictive of post-traumatic growth [27]. In the study of Wu et al. (2021), a positive coping style was confirmed as a determinant of post-traumatic growth in caregivers of people with schizophrenia [62].

The results of the present study also showed that among the dimensions of sense of coherence, the dimension of meaningfulness is able to predict post-traumatic growth. Forstmeier et al. (2009) found that the dimension of the significance of the sense of coherence predicts post-traumatic growth, which was consistent with our results [59]. However, Brockhouse et al. (2011), in a study aimed at determining the effect of sense of coherence, social support and empathy on post-traumatic growth, achieved results inconsistent with the present study [21]. Brockhouse et al. (2011) showed that the sense of coherence has a significantly negative relationship with post-traumatic growth and that the sense of coherence does not predict the post-traumatic growth variable [21]. The results of the study by Silarova et al. (2012) suggested that a sense of coherence predicts physical and mental health in chronic heart patients, which can be somehow in line with the present study [63]. The results of a study by Delgado (2007) showed that a high sense of coherence and spirituality was associated with low stress and high quality of life. Neither sense of coherence nor spirituality had a significant relationship with the objective severity of symptoms. It can be concluded that psychological factors are important in patients’ cognitive interpretation of the disease [64]. The results of the study by Lopez et al. (2014) are in line with the results of the present study, as their study showed that a sense of coherence is effective in improving post-traumatic growth. Religion and life meaningfulness also play a role in predicting post-traumatic growth [58]. The results of the study of Eriksson et al. (2006) are in line with the results of the present study. The results showed that post-traumatic growth is associated with a meaningful life. Explaining the results of the study, it can be stated that people whose lives are meaningful may have a good capacity to link and develop a sense of coherence in stressful situations [65].

### Limitations

One of the limitations of this study is that it is cross-sectional, so the causal relationship cannot be concluded from it. Since sampling was done among the mothers receiving service from Bushehr welfare centers, it is not possible to generalize the results to all mothers with disabled children. Longitudinal studies with more diverse samples are recommended.

## Conclusion

The results of the study indicate that mothers with children with disabilities show some degree of post-traumatic growth, and there is a significant relationship between coping style and sense of coherence with post-traumatic growth. Therefore, improving social and psychological support can be effective in improving the conditions of mothers with children with disabilities. Also, great spiritual changes were observed in these mothers, this demonstrates that how they are influenced by religious beliefs. This spiritual growth is directly related to the mother’s sense of coherence and coping style. Also, emotion-focused coping styles and sub-dimensions of meaningfulness and manageability in the sense of coherence can predict post-traumatic growth. The results of this study clearly show that due to the need of these mothers for government, social and cultural support, it seems necessary to plan and take action to pay more attention to this segment of society.

## Data Availability

The datasets used and/or analyzed during the current study are available from the corresponding author on reasonable request.

## Abbreviations

CSQ: Coping Strategies Questionnaire
CWD: Children with disability
ICF: International Classification of Functioning, Disability, and Health
PTG: Post-Traumatic Growth
SOC: Sense of Coherence

## References

[1] WHO. World report on disability: World Health Organization; 2011. Available from: https://www.who.int/publications/i/item/9789241564182.

[2] Moeini SR, Taraneh MH, Nowroozi R (2015). How to spread disability and its relationship with development Iran. Iran J Official Stat Stud.

[3] Iacolino C, Pellerone M, Pace U, Ramaci T, Castorina V (2016). Family functioning and disability: a study on Italian parents of disabled children. European Proceedings of Social and Behavioural Sciences..

[4] Rabat Jazi AS, Ghafouri A (2014). The Effect of Life Skills educations on the Happiness of Mothers of Children with disability. Special People’s Disabled.

[5] George JE (2017). Challenges Faced by Parents of Children with Learning Disability. J Soc Work Educ Pract..

[6] McConnell D, Savage A, Sobsey D, Uditsky B (2015). Benefit-finding or finding benefits? The positive impact of having a disabled child. Disabil Soc.

[7] Emerson E (2003). Mothers of children and adolescents with intellectual disability: social and economic situation, mental health status, and the self-assessed social and psychological impact of the child’s difficulties. J Intellect Disabil Res.

[8] Lindberg L, Fransson M, Forslund T, Springer L, Granqvist P (2017). Maternal sensitivity in mothers with mild intellectual disabilities is related to experiences of maltreatment and predictive of child attachment: A matched-comparison study. J Appl Res Intellect Disabil.

[9] Kouhsali Ms, Mirzamani SM, Mohammadkhani P, Karimlou M (2007). Comparison of social adjustment between mothers of educable mentally retarded girls and mothers of normal girls in Kashan. Archives of Rehabilitation.

[10] Kamali M (2004). Disability and Human Right. Social Welf Q.

[11] Aftyka A, Rozalska I, Milanowska J (2020). Is post-traumatic growth possible in the parents of former patients of neonatal intensive care units?. Ann Agric Environ Med.

[12] Kiełb K, Bargiel-Matusiewicz KM, Pisula E. Posttraumatic Stress Symptoms and Posttraumatic Growth in Mothers of Children With Intellectual Disability – The Role of Intrusive and Deliberate Ruminations: A Preliminary Report. Front Psychol. 2019;10. 10.3389/fpsyg.2019.02011 PMC6737280 31551869

[13] Kim MY (2017). Factors Influencing Posttraumatic Growth in Mothers of Children With Cancer. J Pediatr Oncol Nurs.

[14] Tedeschi RG, Calhoun LG (1996). The Posttraumatic Growth Inventory: Measuring the positive legacy of trauma. J Trauma Stress.

[15] Zwahlen RA, Rajandram RK, Jenewein J (2016). Post-Traumatic Growth in Oral Cavity Cancer in Relation to Positive Coping Strategies, Hope, and Optimism.

[16] Folkman S. Stress: Appraisal and Coping. In: Gellman MD, Turner JR, editors. Encyclopedia of Behavioral Medicine. New York: Springer New York; 2013. p. 1913–5.

[17] Söderhamn U, Sundsli K, Cliffordson C, Dale B (2015). Psychometric properties of Antonovsky’s 29-item Sense of Coherence scale in research on older home-dwelling Norwegians. Scand J Public Health.

[18] Iqbal N, Sarfaraz A, Aleem S. S B. Sense of Coherence, Social Support and Coping as Predictors of Posttraumatic Growth in Orphan Children. Indian J Posit Psychol. 2013;25(15):123–45.

[19] Xiu D, Mc Gee SL, Maercker A (2018). Sense of Coherence and Posttraumatic Growth:The Moderating Role of Value Orientation in Chinese and Swiss Bereaved Parents. J Loss Trauma.

[20] Linley PA, Joseph S, Loumidis K (2005). Trauma work, sense of coherence, and positive and negative changes in therapists. Psychother Psychosom.

[21] Brockhouse R, Msetfi RM, Cohen K, Joseph S (2011). Vicarious exposure to trauma and growth in therapists: The moderating effects of sense of coherence, organizational support, and empathy. J Trauma Stress.

[22] Tedeschi RG, Calhoun LG. Trauma & transformation: Growing in the aftermath of suffering. Thousand Oaks: Sage Publications, Inc; 1995. p. 163.

[23] Calhoun LG, Cann A, Tedeschi RG, McMillan J (2000). A correlational test of the relationship between posttraumatic growth, religion, and cognitive processing. J Trauma Stress.

[24] Shakespeare-Finch J, Copping A (2006). A grounded theory approach to understanding cultural differences in posttraumatic growth. J Loss Trauma.

[25] Morris BA, Shakespeare-Finch J, Scott JL (2012). Posttraumatic growth after cancer: the importance of health-related benefits and newfound compassion for others. Support Care Cancer.

[26] Fallah R, Keshmir F, Lotfi Kf, Azargashb E, Akbari ME (2012). Post-traumatic growth in breast cancer patients: a qualitative phenomenological study. Middle East Journal of Cancer.

[27] Zhang W, Yan TT, Du YS, Liu XH (2013). Relationship between coping, rumination and posttraumatic growth in mothers of children with autism spectrum disorders. Res Autism Spectr Disorders.

[28] Najafi Gharehasani E, Yazdanbakhsh K, Moment K (2020). Predicting Vicarious Post Traumatic Growth Based on Cognitive Flexibility and Cognitive Reappraisal in Nurses providing Services to Kermanshah Earthquake Victims. J Health Care.

[29] Antonovsky A. Unraveling the mystery of health: How people manage stress and stay well. San Francisco: Jossey-Bass; 1987. p. 218.

[30] Agha Yousefi A, Sharif N (2011). Investigating the Relationship between Sense of Coherence and Emotional Intelligence among Students of Azad University of Tehran. Q J Psychol Stud.

[31] Billings AG, Moos RH (1981). The role of coping responses and social resources in attenuating the stress of life events. J Behav Med.

[32] AraghianMojarad F, Mahmoodi Shan G, Danesh A, Roshandel G (2020). Relationship between Perceived Stress and Coping Strategies of Relatives of Patients Hospitilzed in Cardiac Care Unit. J Nurs Educ.

[33] Heidarzadeh M, Rassouli M, Shahbolaghi F, Alavi Majd H, Mirzaei H, Tahmasebi M (2015). Assessing dimensions of posttraumatic growth of cancer in survived patients. J Holist Nurs Midwifery.

[34] Lu W, Xu C, Hu X, Liu J, Zhang Q, Peng L, et al. The Relationship Between Resilience and Posttraumatic Growth Among the Primary Caregivers of Children With Developmental Disabilities: The Mediating Role of Positive Coping Style and Self-Efficacy. Front Psychol. 2022;12. 10.3389/fpsyg.2021.765530 PMC8764196 35058840

[35] Qin X, Feng Y, Qu F, Luo Y, Chen B, Chen M (2021). Posttraumatic Growth Among Parents of Children with Autism Spectrum Disorder in China and Its Relationship to Family Function and Mental Resilience: A Cross-Sectional Study. J Pediatr Nurs.

[36] Morris JN, Turnbull D, Martini A, Preen D, Zajac I (2020). Coping and its relationship to post-traumatic growth, emotion, and resilience among adolescents and young adults impacted by parental cancer. J Psychosoc Oncol.

[37] Zebrack B, Kwak M, Salsman J, Cousino M, Meeske K, Aguilar C (2015). The relationship between posttraumatic stress and posttraumatic growth among adolescent and young adult (AYA) cancer patients. Psycho-oncology.

[38] Yonemoto T, Kamibeppu K, Ishii T, Iwata S, Tatezaki S-i (2012). Posttraumatic stress symptom (PTSS) and posttraumatic growth (PTG) in parents of childhood, adolescent and young adult patients with high-grade osteosarcoma. Int J Clin Oncol.

[39] Modiri F, Azadarmaki T (2013). Gender and Religiosity. J Appl Sociol.

[40] Mystakidou K, Tsilika E, Parpa E, Kyriakopoulos D, Malamos N, Damigos D (2008). Personal growth and psychological distress in advanced breast cancer. The Breast.

[41] Manne S, Ostroff J, Winkel G, Goldstein L, Fox K, Grana G (2004). Posttraumatic growth after breast cancer: patient, partner, and couple perspectives. Psychosom Med.

[42] Cordova MJ, Cunningham LL, Carlson CR, Andrykowski MA (2001). Posttraumatic growth following breast cancer: a controlled comparison study. Health Psychol.

[43] Nasirzadeh R, Rassolzadeh Tabatabaie K (2009). Religious beliefs and coping strategies in students. Q Horizon Med Sci.

[44] Avaznejad N, Ravanipour M, Bahreini M, Motamed N (2017). Comparison of the Sense of Coherence between Mothers with Healthy Children and Mothers of Children with Chronic Disease in Kerman. J Health Based Res.

[45] Ehteshamzadeh P, Sabri Nazarzadeh R, Memarbashi Aval M (2013). The Relationship Between Sense of Coherence and Job Performance With Intermediation Strategies of Coping with Stress and Mental Health. Psychol Methods Models.

[46] Ghasemi H, Hadianfard H (2014). An Investigation on the Role of Personality Style Vulnerability, Spouse Violence, and Coping Responses in Prediction of Post Partum Depression. J Family Res.

[47] Askari J, Hassanbeigi A, Fallahzadeh H (2011). The rate of various psychological stressors, perceived mental strain due to these stressors, and coping strategies in opium addicts compared to normal individuals. Procedia Social and Behavioral Sciences.

[48] Zandi Karimi A. The effectiveness of group spiritual therapy on resilience and coping styles of cancer patients. Tehran: Alzahra University; 2016.

[49] Lin LY, Orsmond GI, Coster WJ, Cohn ES (2011). Families of adolescents and adults with autism spectrum disorders in Taiwan: The role of social support and coping in family adaptation and maternal well-being. Res Autism Spectr Disorders.

[50] Wilson JZ, Marin D, Maxwell K, Cumming J, Berger R, Saini S (2016). Association of Posttraumatic Growth and Illness-Related Burden With Psychosocial Factors of Patient, Family, and Provider in Pediatric Cancer Survivors. J Trauma Stress.

[51] Taghavi M, Sedighnejad F. Investigating the effect of religion on recovery from post-traumatic stress disorder. The Second National Conference and the First International Conference on New Research in the Humanities. Tehran: Civilica; 2014.

[52] Thombre A, Sherman AC, Simonton S (2010). Religious coping and posttraumatic growth among family caregivers of cancer patients in India. J Psychosoc Oncol.

[53] Prati G, Pietrantoni L (2009). Optimism, social support, and coping strategies as factors contributing to posttraumatic growth: A meta-analysis. J Loss Trauma.

[54] Mehrabi HA, Noroozi S, Mirzaei GR, Kazemi H (2015). An investigation the relationship between post traumatic growth and attachment styles, stress coping styles & quality of life in veterans with post-traumatic stress disorder. J Nurse Physician Within War.

[55] Vollrath M, Torgersen S, Alnæs R (1995). Personality as long-term predictor of coping. Pers Indiv Differ.

[56] Graff RW, Ladd CE (1971). POI correlates of a religious commitment inventory. J Clin Psychol.

[57] Spilka B, Hood RW, Hunsberger B, Gorsuch R (2003). The psychology of religion: An empirical approach.

[58] López J, Camilli C, Noriega C (2015). Posttraumatic growth in widowed and non-widowed older adults: Religiosity and sense of coherence. J Relig Health.

[59] Forstmeier S, Kuwert P, Spitzer C, Freyberger HJ, Maercker A (2009). Posttraumatic growth, social acknowledgment as survivors, and sense of coherence in former German child soldiers of World War II. Am J Geriatric Psychiatry.

[60] Almedom A (2005). Resilience, hardiness, sense of coherence, and posttraumatic growth: all paths leading to “light at the end of the tunnel”?. J Loss Trauma.

[61] Svetina M, Nastran K (2012). Family relationships and post-traumatic growth in breast cancer patients. Psychiatria Danubina.

[62] Wu C, Liu Y, Ma S, Jing G, Zhou W, Qu L (2021). The mediating roles of coping styles and resilience in the relationship between perceived social support and posttraumatic growth among primary caregivers of schizophrenic patients: a cross-sectional study. BMC Psychiatry.

[63] Silarova B, Nagyova I, Rosenberger J, Studencan M, Ondusova D, Reijneveld SA (2012). Sense of coherence as an independent predictor of health-related quality of life among coronary heart disease patients. Qual Life Res.

[64] Delgado C (2007). Sense of coherence, spirituality, stress and quality of life in chronic illness. J Nurs Scholarsh.

[65] Eriksson M, Lindstrom B (2006). Antonovsky’s sense of coherence scale and the relation with health: a systematic review. J Epidemiol Commun Health.

